# Current status of evaluation and treatment of early-stage remnant gastric cancer

**DOI:** 10.3389/fonc.2024.1457564

**Published:** 2024-11-13

**Authors:** Yinghui Huang, Li Ma, Keyu Ren, Qun Gao, Zhenming Zhu, Guangrong Wang, Bin Cao, Baoguo He

**Affiliations:** ^1^ Department of Gastroenterology, The Affiliated Hospital of Qingdao University, Qingdao, China; ^2^ Department of Nutrition, The Affiliated Hospital of Qingdao University, Qingdao, China

**Keywords:** remnant gastric cancer, early gastric cancer, endoscopic submucosal dissection, duodenogastric reflux, endoscopic surveillance, subtotal gastrectomy

## Abstract

Because of early diagnosis and improved prognosis, patients with gastric cancer are now surviving longer and remnant gastric cancer after gastrectomy is becoming more common. Remnant gastric cancer is traditionally considered a malignancy with a dismal outcome. However, recent advances in diagnostic and therapeutic strategies have improved outcomes. In recent years, the development of endoscopic therapy has provided us with new therapeutic ideas. Although with some drawbacks, such as limited working space, gastric fibrosis and staples under the suture line, endoscopic management is still an effective treatment option with potential long-term survival advantage. For gastrectomy patients, endoscopic surveillance should also be recommended, for prompt surveillance and detection in the early remnant gastric cancer. This review aims to provide an overview of remnant gastric cancer, especially focusing on its pathogenesis as well as new advances in the diagnosis and endoscopic treatment of early-stage remnant gastric cancer.

## 1 Introduction

Gastric cancer is the fifth most common cancer and the third leading cause of cancer death worldwide (1). Risk factors for the disease include H. pylori infection, age, high salt intake, and a low diet of fruits and vegetables (2). Among the types of gastric tumors, remnant gastric cancer (RGC) is usually defined as a tumor that develops in the gastric remnant more than 5 years after a previous gastrectomy and is generally associated with a worse prognosis (3). It can occur either after a previous resection for benign or malignant lesions, and its incidence is reported to comprise 1%−8% of all GC (4). While, the incidence of MMGC (metachronous multiple gastric cancer) was 2.4% (5). As patients with gastric cancer are now surviving longer because of early diagnosis and improved prognosis, remnant gastric cancer after gastrectomy for malignant disease is becoming more common, especially in Eastern countries (6). Furthermore, recent advances in diagnostic and therapeutic techniques have contributed to early detection and the minimally invasive treatment of RGC.

## 2 Definition

Remnant gastric cancer (RGC) was originally defined as the gastric cancer detected more than 5 years after a distal gastrectomy for benign disease, which was first described in 1922 (7). In 1982, the Japanese Gastric Cancer Association organized the first national cross-sectional study on remnant gastric cancer, and the results showed that 10 years was the optimal point for distinguishing between new and recurrent cancers, regardless of whether the gastric cancer was in the early or progressive stage at the time of surgery (8). Based on this, the Japan Society for the Study of Postoperative Gastric Complications (JSSPC) conducted a retrospective study of pathological data from a national questionnaire survey on the platform of a national academic organization and defined remnant gastric cancer as new-onset cancer appearing in the remnant stomach more than 10 years after gastric cancer surgery (9). In 1998, the Japanese Society for Gastric Cancer adopted the concept of “cancer on the remnant stomach”, which first appeared in the 13th (Japanese) and 2nd (English) editions of the Japanese Gastric Cancer Treatment Guidelines (10). It does not differentiate between the nature of gastric disease, extent of resection, and reconstruction modality for the first gastric surgery, and does not bind to a specific time interval. The main reason for the establishment of this conception is that it is difficult to distinguish between remnant and recurrent gastric cancer due to the difficulty in evaluating the recurrence and exclusion factors of gastric cancer, and it is controversial to define remnant gastric cancer only in terms of the time interval after the first operation. Chinese surgeons’ consensus opinion for the definition of gastric stump cancer is that at the present stage of clinical work, new cancers appearing in the remnant stomach more than 5 years after gastrectomy for benign diseases or more than 10 years after gastrectomy for gastric cancer are more in line with the definition of remnant gastric cancer in China (11).

## 3 Risk factors

Remnant gastric cancer can be divided into two categories: gastric stump carcinoma and metachronous multiple gastric cancer. Gastric stump carcinoma is gastric cancer that occurs after gastric resection for benign disease, and metachronous multiple gastric cancer occurs in the remnant stomach after gastric cancer surgery. The risk factors of these two types are different. The factors involved in metachronous multiple gastric cancer are not very different from those involved in carcinogenesis in unresected stomachs. The factors involved in gastric stump carcinoma differ from those involved in unresected stomachs. We will provide a detailed introduction to the risk factors of gastric stump carcinoma.

Reflux of bile juice to remnant stomach is a major factor in the occurrence of gastric stump carcinoma (12). A systematic literature review comprising a total of 21 studies found that IM individuals are much easier to develop GC compared to those without IM (13). Bile reflux can induce intestinal metaplasia(IM),which is the inevitable precancerous stage to develop intestinal-type gastric cancer (GC) (14). Deoxycholic acid (DCA) is the main bile acid (BA) component of duodenogastric reflux.

In Duochen Jin. et al.’s study, exposure to DCA can activate a novel signaling axis comprising TGR5-STAT3-KLF5 in the gastric epithelium (15). In gastric epithelial cells, DCA promoted proliferation and apoptotic resistance, upregulated proinflammatory cytokines and IM markers, and facilitated STAT3 phosphorylation, nuclear accumulation and DNA binding to the KLF5 promoter, which promotes the occurrence of cancer. This tumor-promoting activity of bile acids was also demonstrated in rat gastric carcinomas induced by N-methyl-N’-nitro-N-nitrosoguanidine (16–18). Several bile acids stimulate replicative DNA synthesis and induce the activity of ornithine decarboxylase in the antral mucosa of the glandular stomach in rats (19).

In cases of duodenogastric reflux, bacteria enter the stomach under physiological conditions and live in the lumen of the duodenum. Colonization of the mucosal surface with bacteria that are not characteristic of the gastric microbiota induces inflammation in it, which enhances the pathogenic effect of the bile acids themselves (20). Progressive BG can lead to atrophy of the gastric mucosa, intestinal metaplasia, epithelial dysplasia and eventually to gastric cancer (21, 22). In Matsuhisa T. et al. ‘s study, they demonstrated that the development of intestinal metaplasia due to exposure to high concentrations of fatty acids does not depend on the H. pylori (23). In addition, under the influence of fatty acids and their salts, even after successful eradication therapy, the function of the mucosal barrier remains impaired with a change in the microRNA profile (24). The distal gastrectomy-induced achlorhydria promotes the growth of microorganisms in the remaining stomach, per Correa’s idea (25). Nitrate in saliva and food is converted to nitrite by nitrate-reducing bacteria. Carcinogenic N-nitroso compounds can be created when nitrite combines with amines or amides in an acidic environment or when bacteria catalyze the reaction. Bile acids may be potential amides in intragastric nitrosation, and the remaining stomach exhibits significant duodenal reflux (26). Indeed, N-nitroso-bile acids such as N-nitroso-glycocholic acids and N-nitroso-taurocholic acids are mutagenic and carcinogenic (27). Additionally, this team’s recent work showed that giving rats oral thioproline, an efficient nitrite-trapping agent, stopped the development of stomach cancer brought on by duodenogastric reflux (28). Therefore, endogenous nitrosation after Billroth II gastrectomy will be a key investigation to resolve the mechanism of gastric stump carcinogenesis.

Analysis of data from a countrywide Japanese survey revealed significant differences in the distribution of the types of gastrectomy or reconstruction surgeries based on the time interval between the first gastrectomy and therapy for MMGC (29). Respectively. Twenty-two percent (103/462) of patients who had surgery for MMGC within ten years after their first DG had B-II, but only eight percent (23/286) of those who had surgery for MMGC within five years had B-II. On the other hand, R-Y only explained 1.3% (6/462) of patients who had MMGC surgery within 10 years after their original DG and 21.7% (65/286) of patients who had MMGC surgery within 5 years. Similarly, the occurrence of gastric stump carcinoma after different surgical methods can also be explained by bile reflux. A meta-analysis conducted by Tersmette et al. demonstrated that the incidence of remnant gastric cancer at 15–20 years after B-II was significantly higher than that after B-I (30). There are a large number of experiments which have proved the Billroth II procedure induces bile reflux more easily: Lindecken et al. (31) examined the residual stomach using hepatobiliary sequence scintigraphy and identified bile reflux in 53% of patients after B-II anastomosis. Furthermore, in a recent randomized controlled trial, the bile reflux of B-II occurrence significantly increased compared with of uncut RNY one year postoperatively (32). Still, a study from Italy (33) and a recent retrospective database review (34) demonstrated that type of reconstruction did not affect the risk of newly developed RGC. From these findings, whether B-II reconstruction results in a higher risk of newly developed RGC than other reconstruction remains uncertain. Therefore, further research is needed on whether reconstruction types after distal gastrectomy affect the incidence of gastric stump carcinoma.

Furthermore, the increased dietary fat plays an important role in the etiology of gastric stump carcinoma. The experiment shows that rats which received Billroth II gastrectomy have gastric stump carcinoma 50 weeks after surgery while those which received Billroth I do not have any carcinomas (35). Since the high-fat diet stimulated bile excretion into the feces in the present experiment, which also supports that bile is the responsible factor for gastric stump carcinogenesis.

Moreover, Epstein-Barr Virus (EBV) infection is also one of the risk factors of gastric stump carcinoma. Stomach inflammation and atrophy in gastric epithelial cells due to long-established H. pylori infection may attract B lymphocytes harboring latent EBV, initiating B cell lytic cycle, and facilitating viral transmission (36). EBV may increase the risk of malignant transformation in the stomach through microRNAs such as BARF1, which are strongly expressed in gastric cancer cells and have been shown to act as oncogenes, promoting cell proliferation by upregulating transcription factor signals and reducing cell cycle inhibitors (37–39). DNA methylation is also crucial to the oncogenetic process (40), induced by EBV LMP2A (latent membrane protein 2A), often expressed in EBV-associated gastric cancer (39).

Gastric mucosal blood flow, secretion of mucin, and renewal of the gastric mucosal cells are considered to be defensive factors against gastric mucosal injuries. These factors are regulated by the nervous system and neuropeptides. Gastrectomy may affect this regulation and induce gastric mucosal changes, such as atrophic gastritis and carcinoma. In M Kaminishi. et al. ‘s study, the effect of gastric mucosal denervation on residual gastric tumorigenesis was investigated. After gastrectomy, not only duodenogastric reflux, but also the denervation of the gastric mucosa play an important role in the etiology of gastric remnant cancer (41). Some epidemiological studies also suggest that vagotomy can increase the risk of gastric cancer development. In one of them, the authors found an increased risk of gastric cancer development 3.5 times higher than expected comparing patients underwent to a vagotomy for treatment of peptic ulcer and controls (42). In another study, which examined cancer incidence in 1,992 patients undergoing to a gastric surgery found that, particularly, the vagotomized had a significantly increased risk of gastric cancer (43). Some experimental studies also suggest that vagotomy can raise the risk of developing gastric cancer: in one study the author refers to be possible to obtaining gastric cancer in rats subjected to a truncal vagotomy and drainage procedure without using carcinogens; in another study was reported that vagotomy significantly increases the incidence and number of gastric adenocarcinoma obtained from rats treated with MNNG; and finally one study reports that vagotomy associated with duodenogastric reflux in rats treated with MNNG increases the number of tumors obtained when compared to duodenogastric reflux only, and believe that denervation of the gastric mucosa not only leads to decreased of gastric mucosa cell function and cytoprotection but it also increases the regeneration of immature cells (44). Accordingly, clinical studies demonstrate that patients with gastric ulcers who have undergone a vagotomy have a greater risk of stomach, colorectal, biliary tract, and lung cancers (45, 46).

## 4 Pathology

Numerous studies have described the clinical features of remnant gastric cancer. However, little is known regarding the histological features of the remnant gastric cancer.

Firstly, as for the location of the tumor, no significant difference was observed in the pathological type, histological subtype, Borrmann type, tumor differentiation, and TNM stage between patients with GC in the greater curvature and those with GC in the lesser curvature (47). Prashanth Sangu et al. (48) shows that GSC tends to originate from the anastomotic site regardless of the primary disease. Lee et al. (49) and Ojima et al. (50) reported that tumor location did not significantly affect survival. In contrast, Firat et al. (51) stated that tumor location at the anastomotic site is possibly a good prognostic factor. On the other hand, Namikawa et al. (52) found that patients with tumors in the anastomotic site had a poor prognosis. Therefore, the importance of tumor location for survival is still controversial.

Secondly, among prognostic factors, tumor invasion depth was the only independent factor affecting RGC’s long-term outcome. The clinicopathological factors that were generally associated with the long-term outcome of the RGC patients were undifferentiated type, with vascular invasion and serosal or other organ invasion (49, 53, 54). However, during the multivariate Cox regression analysis of Kenichi Iwasaki et al., it has showed that tumor invasion depth was the only independent prognostic factor for RGC patients (55). Moreover, Komatsu et al. and Li et al. found no significant difference in the survival rate between the RGC and the initial cancer and in the outcome of the RGC from that of the initial cancer in the same region (e.g., cancer in the cardiac or pyloric region) (54, 56).

## 5 Diagnose

**Figure 1:**
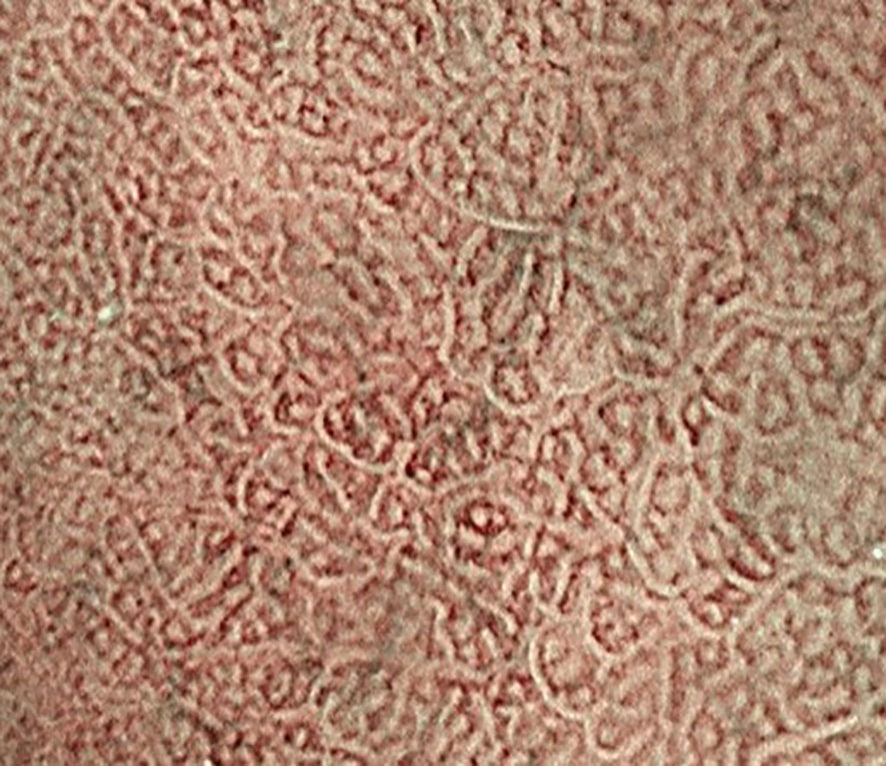
Compared with those in the surrounding tissue, the signal in the mucosal capillaries was lower. The white zone reflects the morphology of the marginal crypt epithelium.

Early GSC lacks specific clinical symptoms, and some patients may have gastrointestinal dysfunction similar to postgastrectomy syndrome, which is easily mistaken for ulcer recurrence. The most common first symptoms of RGC are epigastric discomfort and body weight loss, the tumor is located in the proximal 1/3 of the stomach or the gastroesophageal junction is often accompanied by swallowing discomfort, choking, and located at the anastomotic mouth will have nausea, vomiting, obstruction and other manifestations. Early GSC has no specific symptoms, so endoscopic screening is extremely important. Considering that the mucosa of remnant stomach is swollen, and the tumor margins are difficult to judge. the remnant stomach couldn’t be inflated easily. To this end, the development and advancement of endoscopic equipment greatly contributed to endoscopic diagnosis of gastric tumors. In particular, the development of image-enhanced endoscopy, represented by narrow band imaging (NBI) (**Figure 1**), has dramatically improved the qualitative and quantitative diagnosis of gastrointestinal tumors. Because of the two wavelengths in narrow band light, the contrast in the surface is enhanced, and the operators can observe the surface and vascular pattern clearly (57). Furthermore, when used in combination with magnifying endoscopy, the mucosal capillaries and glandular structures can be evaluated in more detail (58). What’s more, it may be possible to try endoscopic ultrasonography (EUS) examination for diagnosing the tumor depth more precisely (59). The results could be same in GSC.

It’s believed that the simultaneous use of CT and gastroscopy imaging may help to accurately diagnose the T stage of GSC preoperatively. While the diagnosis of T1/2 residual gastric cancer must be carefully considered due to the high rate of misdiagnosis.

What’s more, a team in China has developed an AI system, ENDOANGEL‐LD, a deep learning algorithm with retrospective and real time endoscopic images which has been demonstrated to exhibit a sensitivity and specificity of over 90% in detection of early gastric when trialed prospectively in over 2000 patients (60). These advancements in deep learning algorithms could provide better framework of future models to aid endoscopists in detection of early remnant gastric cancer.

## 6 Treatment

### 6.1 Transabdominal surgery

The mainstay of treatment for RGC patients is radical surgical resection during the passing few years. It involves removing the entire remnant stomach with lymph node dissection; this is known as a completion total gastrectomy with lymph node dissection.

R0 resection is an important prognostic factor in RGC, as well as conventional gastric cancer (61). However, the optimal surgical treatment has not been fully established, especially in the field of lymph node dissection. Benign disease without lymph node dissection may have the same incidence of lymphatic flow and metastasis after the initial gastrectomy as in primary gastric cancer. Lymph node metastases of previous initial malignant disease can occur in anomalous stations, mainly in the jejunal mesentery after Billroth II (BII) reconstruction, as observed in Asian studies (62). The alteration in gastric lymphatic drainage is one of the hypotheses that justifies the apparent increase in neoplastic recurrence among more advanced stages of RGC.

Gastrectomy was mostly performed as an open procedure (OG), but more recently, minimally invasive surgical (MIS) approaches have undergone widespread adoption, including laparoscopic-assisted gastrectomy (LAG) and robotic-assisted gastrectomy (RG) (63). Compared with the open surgery, MIS is advantageous as patients tend to be subject to less physiological stress, immunologic burden, faster recovery times, lower complication rates, and less immediate and long-term burden on healthcare resources (64, 65).

Laparoscopy provides a magnified view of minute structures such as tiny vessels and nerves, which allows lymphadenectomy to be more precisely performed, potentially leading to less intraoperative blood loss and fewer postoperative complications (66). Ryuhei Aoyama found that there was no difference in the overall rates and patterns of recurrence between MIS and OG, suggesting that the surgical approach did not affect disease recurrence (67). But one thing to notice that Indications for MIS were limited to no evidence of serosa invasion or lymph node metastasis to extraperigastric areas on preoperative evaluation (6).

The other choice for MIS is RG, which is more advanced. SRG (RG performed without the assistant’s laparoscopic forceps assistance) for gastric cancer was technically feasible and effective with favorable short-term outcomes, including shorter operative time, less estimated blood loss, shorter hospital stays, and lower postoperative morbidity than those in LG (68). What’s more, Rana M Alhossaini’s survey shows that the robotic approach demonstrated a lower conversion rate than laparoscopy, although the statistical difference was marginal (69).

In conclusion, all the results suggest that proficiency in advanced laparoscopic surgical techniques, such as proper adhesiolysis and stable laparoscopic anastomosis, will allow laparoscopic gastrectomy for remnant gastric cancer to be performed with satisfactory short-term results. This minimally invasive approach can be one treatment option for remnant gastric cancer (70).

The standardization of RGC surgery and the development of multiple adjuvant treatment therapies have improved the survival rate of RGC patients, but the outcomes of surgery for GRC remain variable, with 5-year survival rates ranging from 23.1% to 95% (55, 71–73), and RGC patients may relapse after surgery. Normally, postoperative follow-up is applied to surveil relapse of RGC. However, due to the rarity of RGC, there is still no specialized follow-up strategy for RGC nearly a century after it was first described. One study from China (74) established a follow-up model for patients with RGC, which recommend focusing on postoperative follow up for the first 3 years after RGC diagnosis and setting different follow-up intensities and frequencies (3-6 months) according to different stages: For patients with stage I disease, follow‐up visits are recommended every 6 months for the first 3 years after surgery. In the 4th and 5th years after surgery, follow‐up visits are recommended once annually. For patients with stage II‐III disease, follow‐up visits are recommended every 3 months in the first 2 years after surgery, every 6 months in the 3rd year after surgery, and annually in the 4th and 5th years after surgery. For RGC patients who have survived for more than 5 years after surgery, the visits should be based on routine healthcare programs and supplemented by specialist examinations related to GC. This study may give a hand to determining how to conduct effective prognostic risk stratification for such rare patients and develop a personalized follow-up strategy in the future.

### 6.2 Endoscopic treatment

Endoscopic resection is first-line therapy in the management of superficial neoplasms throughout the gastrointestinal tract, as well as an increasingly viable therapeutic alternative in the resection of selected small deep lesions throughout the upper and lower gastrointestinal tract. The mainstay of therapy has traditionally been endoscopic snare polypectomy and endoscopic mucosal resection. Moreover, recent innovative advancements took place in therapeutic endoscopy (75). Endoscopic mucosal resection (EMR) and endoscopic submucosal dissection (ESD) are two new well-known endoscopic resection procedures used for advance gastrointestinal lesions. As compared to standard polypectomy techniques, EMR and ESD provide wider and deeper resection margins and allow en bloc removal of lesions for more detailed pathology with curative intent for early neoplastic gastrointestinal lesions.

In the past, remnant gastric cancer (RGC) was commonly detected at an advanced stage that radical surgical resection has previously been considered to be the only method for achieving cure of RGC. However, it was found that completion gastrectomy does not improve survival outcomes compared with endoscopic resection and it may even adversely affect the long-term outcomes of patients with ERGC.

A noteworthy result in Yudai Fukui’s study was that completion gastrectomy was adversely correlated with survival outcomes. Although cancer-related death was not observed in the current population, completion gastrectomy was associated with sixfold higher risk of death compared with the endoscopic management. The reason of poor prognosis after completion gastrectomy is unclear. However, given that the median age of the current population exceeded 70 years and three of seven deceased cases in surgery group died from respiratory complications directly or indirectly related to decreased performance status and/or respiratory capacity, malnutrition status caused by completion gastrectomy might affect the survival outcomes. Accordingly, the indication of completion gastrectomy should be carefully determined even in the cases with suboptimal histopathological findings after endoscopic resection considering the long-term survival risk and oncological benefits (76).

In a latest meta-analysis, it had been found that ESD for early gastric neoplastic lesions had high en bloc, complete and curative resection rates (0.93,0.84,0.78), similar to previously published outcomes in the unaltered stomach (77). As for the analysis of adverse events, the bleeding rates are similar, however, the perforation rates were slightly higher in the surgically altered stomach, especially in the gastric tube when compared to results of previously published cohorts. Furthermore, perforation remains one of the most crucial complications of ESD, whether it is performed in the normal or surgically altered stomach (78). Perforation in the remnant stomach readily causes peritonitis due to the reflux of duodenal content containing bile and pancreatic juice. Therefore, a rapid endoscopic treatment for perforation, including clips, polyglycolic acid sheet with fibrin glue, or Over-The-Scope Clip, is essential for minimizing adverse events.

In the last few years, several studies have been conducted to confirm the oncological feasibility of providing endoscopic treatment in patients with gastric stump carcinoma. The 5-year overall survival rate was 71.0~88.4%, and the 5-year gastric cancer-specific survival rate was 97.6~100% (79–81) (**Table 1**).

**Table 1:** Clinical outcomes of endoscopic submucosal dissection for remnant gastric cancer.

Ref.	No. of patients	No. of ESD lesions	En bloc resection	Complete resection	R0 resection	Curative resection	Perforation	Bleeding	Recurrence	5-yr OS	5-yr DSS
Tsuda et al., 2023 (80)	256	270	94.90%	NA	86.70%	NA	NA	NA	3.13%	81.3%,	98.10%
Murakami et al., 2022 (79)	25	27	88.90%	/NA	85.20%	NA	0	0	4%	71%	100%
Liu et al., 2022 (87)	48	NA	100%	NA	91.70%	73.90%	0	10.40%	10.40%	NA	91.30%
Kim et al., 2020 (88)	25	NA	92%	NA	84%	NA	NA	NA	NA	NA	NA
Yabuuchi et al., 2019 (81)	157	165	95.50%	NA	84.70%	66.90%	11.50%	9.60%	8.28%	88.40%	97.60%
Nomura et al., 2018 (89)	NA	138	NA	89.10%	NA	77.50%	2.20%	4.30%	NA	NA	NA
Song et al., 2017 (90)	29	31	90%	77%	71%	/	3%	6%	0	96.60%	100%
Ojima et al., 2016 (91)	NA	34	100%	85.30%	NA	NA	NA	NA	NA	58.40%	96.20%
Lee et al., 2016 (92)	18	NA	88.90%	NA	100%	91.70%	5.56%	0	0	NA	NA
Yamashina et al., 2015 (93)	42	42	NA	NA	NA	NA	0	4.80%	NA	81.80%	NA
Tanaka et al., 2014 (84)	32	33	100%	NA	93.90%	NA	9.38%	3.13%	NA	NA	NA
Ojima et al., 2014 (94)	NA	49	100%	85.70%	NA	81.60%	12.20%	2%	NA	NA	NA

NA, Not applicable; ESD, endoscopic submucosal dissection; 5-yr OS, 5-year overall survival; 5-yr DSS, 5-year disease-specific survival.

ESD also has obvious drawbacks. ESD for EGC in the remnant stomach after gastrectomy is technically even more demanding because of the limited working space in which to perform the procedure as well as the presence of severe gastric fibrosis and staples under the suture line, leading to poor outcomes of ESD.

However, removal of surgical staples may help secure a better endoscopic view and allow for more reliable ESD in patients with residual gastric cancer in the remnant stomach or gastric conduit by reducing the risk of specimen damage, increasing the procedure speed, and eventually allowing for complete and curative resection without complications (82).

Given that staples in the suture line pose a risk of perforation, most surgeons aim to dissect directly above the staples. However, a shallower dissection layer can damage the specimen, leaving behind tumor remnants. On the other hand, dissecting below the staples poses a risk of perforation. Furthermore, dissection in the layer containing the staples results in a continuous flow of current through the staple, increasing the time required for the dissection or possibly increasing the risk of delayed perforation.

For these reasons, appropriate devices should be selected according to the situation during ESD (83). Shinwa Tanaka considers that the Flush Knife-BT can help overcome limited space thereby enable more precise manipulation. In cases of severe fibrosis, they used the ST hood and a 1.0-mm-long Flush Knife, which may lead to the achievement of en bloc resection in all cases in the anastomotic group in their study (84). Additionally, according to the Yugo Suzuki’s research, the Dual Knife could be another good choice. The Dual Knife was used to conduct EndoCut electrosurgical current into the staple. The current through the staple results in an EndoCut effect on the tissue that is in contact with the staple, which will then release the staple if some tension is placed. This could have also been achieved by grasping the suture with coagulation forceps or any other knife (eg, hook knife: KD-620QR; Olympus) while applying EndoCut current. Removal of the staples allowed us to secure a better endoscopic view and more reliable ESD, which resulted in safer treatment (83).

**Figure 2:**
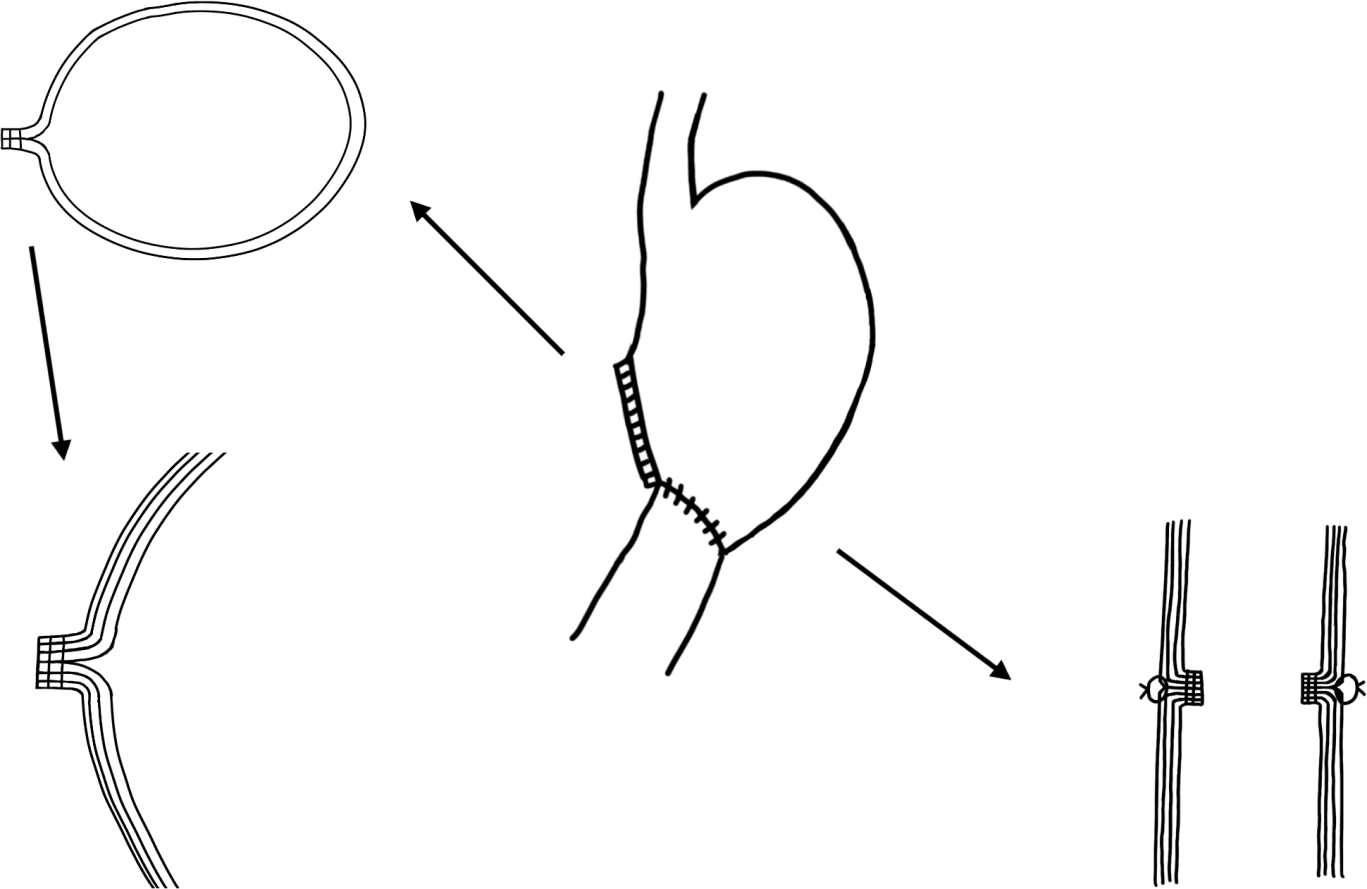
The structural relationship between the gastric mucosa and muscularis after distal gastrectomy with sutures in China.

To remove surgical staples, it is also very important to understand the anatomical structure of the suture line resulting from the previous gastrectomy. With the development of science and technology, the main anastomosis of gastrectomy has changed from manual suture to mechanical anastomosis. There are two main types of stapling machines for gastrectomy: the Linear cutter and the Purse-string device. Linear Cutter was used to cut and suture the stomach, while the Purse-string Device was used for the gastrointestinal anastomosis (**Figure 2**). The staples of the Purse-string Device are located in the lumen of the digestive tract, which makes it feasible to remove the same level of tissue, or the tissue beneath staples during ESD, but the resection line has to be established at the same level or just above the staples when the Linear cutter is used. When staples are clearly recognizable, it is acceptable to remove such staples by using forceps to avoid interfering with the actual dissection procedure (85).

As confirmed in the multivariate analysis, pT status and lymphatic invasion were identified as independent risk factors for LN metastasis in RGC (86). Given the minimal risk of lymph node involvement in ERGC confirmed in several studies, oncological significance of regional lymphadenectomy for ERGC may be limited. In Yudai Fukui’s study, they analyzed the efficacy of endoscopic management for ERGC. The results suggest that endoscopic management would be sufficient for ERGC even in cases presenting histopathologically “noncurative” features if lymphovascular infiltration is not confirmed in the pathologic specimen. Noncurative endoscopic resection was not always associated with tumor recurrence even in the cases with histopathologically positive resection margins or large, deeply infiltrative tumors (76). In addition, lymphatic drainage in the remnant stomach might be changed after previous gastrectomy, and regional lymph node may have already been deprived when the previous surgery was performed for malignant disease. Therefore, the necessity of completion gastrectomy for securing the en bloc resection of regional lymph is doubtful.

In conclusion, endoscopic management is an effective treatment option for ERGC with potential long-term survival advantage over the conventional radical surgery. Additional surgical resection might be avoided in selected cases that can even present noncurative features after endoscopic resection if macroscopic complete resection is achieved and lymphovascular infiltration is not observed.

## 7 Conclusion

Gastric stump cancer will not remain a rare clinical problem and may be more frequently encountered in the future. This entity still needs introspection and research concerning precise definition, appropriate staging and management. Owing to recent advances in diagnostic and therapeutic options, gastric stump carcinoma can be detected early and get timely treated. Endoscopic management and minimally invasive surgery feasible in selected patients may offer a better quality of life.
